# The complete mitochondrial genome sequences of *Nanorana chayuensis*

**DOI:** 10.1080/23802359.2020.1715871

**Published:** 2020-01-22

**Authors:** Min Tang, Jiahong Liao, Liqing Peng, Lichun Jiang, Zhangqiang You, Wei Chen

**Affiliations:** Ecological Security and Protection Key Laboratory of Sichuan Province, Mianyang Normal University, Mianyang, China

**Keywords:** Dicroglossidae, *Nanorana chayuensis*, mitochondrial genomes

## Abstract

In this study, we obtained the complete mitochondrial genome sequence of *Nanorana chayuensis.* The mitogenome length is 17,882 bp, including 13 protein-coding genes (PCGs), 22 transfer RNA genes (tRNA), 2 ribosomal RNA genes (rRNA), and 1 non-coding control region (CR). Present data will contribute to further analysis of phylogenetic relationship and population genetics of this species.

*Nanorana chayuensis* is distributed in Tibet and Yunnan China, and lives in the stream with an altitude of 1000–1540 m (Fei et al. 2012). Its population is declining due to habitat fragmentation (Fei et al. 2012). In this study, we collected the sample of *N. chayuensis* in Chayu County China (28°30′11.01″N, 97°0′59.68″E; 1532 m in altitude) and sequenced its complete mitochondrial genome using Illumina high-throughput sequencing method. The specimen was kept in the Museum of Life Science and Technology of Mianyang Normal University (specimen code MNU20190419).

The complete mitochondrial genome of *N. chayuensis* (GenBank accession no. MN411630.1) is 17,882 bp including 13 PCGs, 22 tRNA genes, 2 rRNA genes, and 1 control region, which is similar to the mitochondrial composition of most vertebrates (Jiang et al. 2016, 2019). The overall base composition is A: 29.1%, C: 25.6%, G:14.4%, T: 31.0%, and the content of G + C base in the mitochondrial genome of *N. chayuensis* is 40%. Except for tRNA-Pro, tRNA-Gln, tRNA-Ala, tRNA-Asn, tRNA-Cys, tRNA-Tyr, tRNA-Ser, tRNA-Gly, and ND6 genes, most genes are encoded on the heavy strand. Among the 13 protein-coding genes in the mitochondrial genome, 6 genes (COXII, ATP8, ND4L, ND5, ND6, Cytb) share the starting codon ATG, GTG is the starting codon of 3 genes (ND1, ND3 and COX1), ND2 and ND4 use ATT as the starting codon, ATP6 and COXIII use the starting codon ATA. Six genes (ND1, COXII, COXIII, ND2, ND3, and ND4) are inferred to terminate with an incomplete stop codon T––. ND4L and Cytb share the termination codon TAG. TAA is the termination codon of ATP6, ATP8, and ND5. COX1 and ND6 have stop codon AGG and AGA, respectively. The total length of 22 tRNA genes was 1613 bp with individual gene size ranging 66–74 bp. 12S rRNA (935 bp) and 16S rRNA (1590 bp) are located between tRNA-Phe and tRNA-Leu, separated by tRNA-Val gene. There are totally 41 bp overlapped nucleotides between adjacent tRNA genes in 11 locations, and the length of the overlapped sequence is 1–15 bp. While there are totally 76 bp intergenic nucleotides in 11 locations, the length of intergenic spacer is 1–28 bp. The non-coding region (2315 bp) is bound by tRNA-Phe and Cytb. The NJ phylogenetic tree (Figure 1) of Dicroglossidae was made, and *N. chayuensis* showed close relationship with other *Nanorana* species.

**Figure 1.**
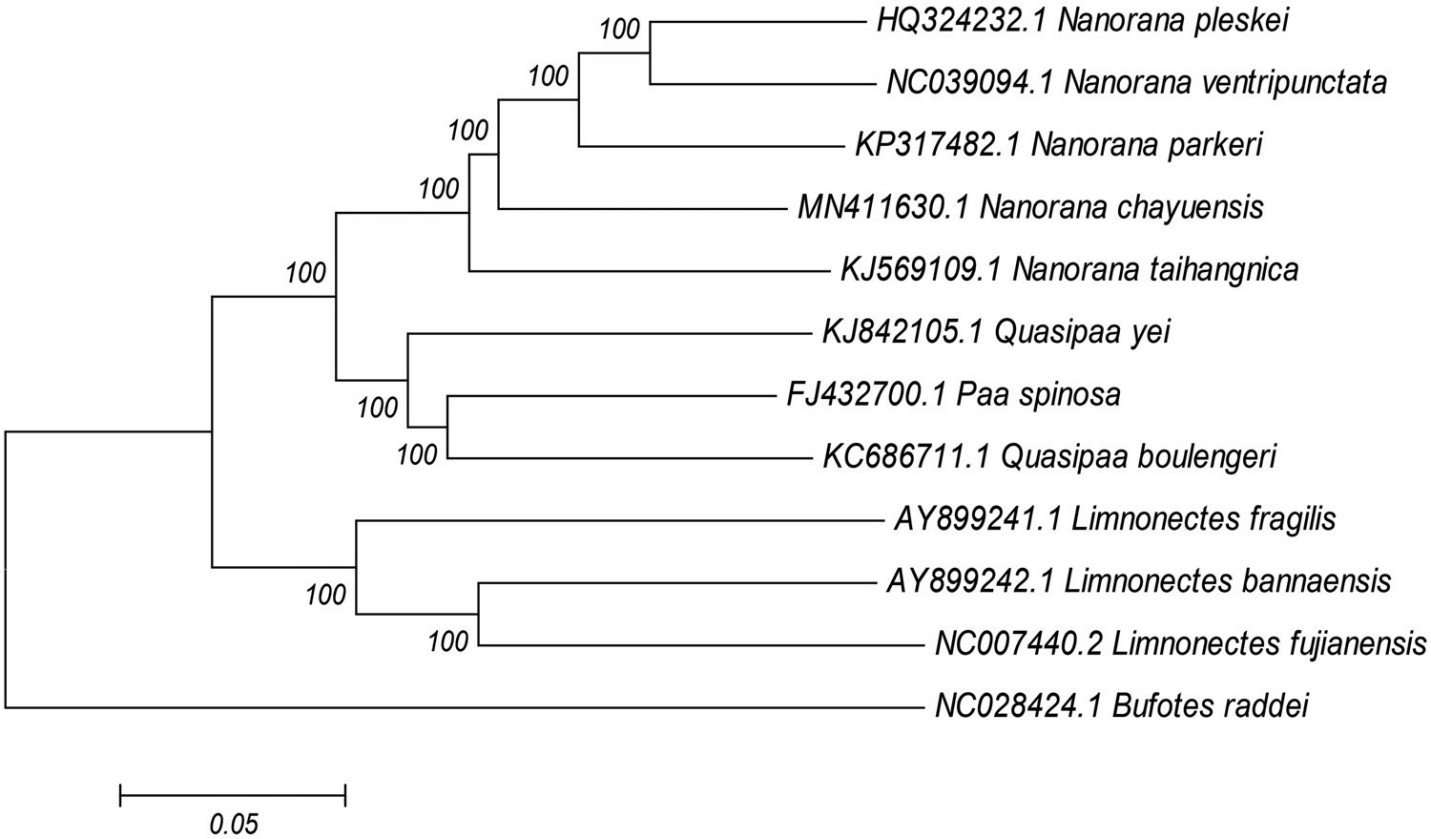
Neighbor-joining phylogenetic tree of Dicroglossidae representatives produced based on complete mitochondrial genomes. Genbank accession numbers and bootstrap values of nodes are shown on the tree.

In this study, the whole nucleotide sequence of the mitotic genome of *N. chayuensis* was determined, and these basic data will contribute for the further analysis of phylogenetic relationship and population genetics of Dicroglossidae species.

## References

[CIT0001] Fei L, Ye CY, Jiang JP. 2012. Colored atlas of Chinese amphibians and their distributions. Chengdu: Sichuan Publishing House of Science and Technology.

[CIT0002] Jiang LC, Peng LQ, Tang M, You ZQ, Zhang M, West A, Ruan QP, Chen W, Merilä J. 2019. Complete mitochondrial genome sequence of the *Himalayan Griffon*, *Gyps himalayensis* (Accipitriformes: Accipitridae): Sequence, structure, and phylogenetic analyses. Ecol Evol. 9(15):8813–8828. 31410282 10.1002/ece3.5433 PMC6686361

[CIT0003] Jiang L, Ruan Q, Chen W. 2016. The complete mitochondrial genome sequence of the Xizang Plateau frog, *Nanorana parkeri* (Anura: Dicroglossidae). Mitochondrial DNA Part A. 27(5):3184–3185. 10.3109/19401736.2015.1007327 25758045

